# Functional neuroplasticity of facilitation and interference effects on inhibitory control following 3-month physical exercise in aging

**DOI:** 10.1038/s41598-024-53974-5

**Published:** 2024-02-14

**Authors:** Hong-Yi Wu, Chih-Mao Huang, Ai-Ling Hsu, Chiao-Nan Chen, Changwei W. Wu, Jyh-Horng Chen

**Affiliations:** 1 Graduate Institute of Biomedical Electronics and Bioinformatics, National Taiwan University, Taipei, Taiwan; 2 Department of Biological Science and Technology, National Yang Ming Chiao Tung University, Hsinchu, Taiwan; 3 Bachelor Program in Artificial Intelligence, Chang Gung University, Taoyuan, Taiwan; 4 Department of Psychiatry, Chang Gung Memorial Hospital at Linkou, Taoyuan, Taiwan; 5 Department of Physical Therapy and Assistive Technology, National Yang Ming Chiao Tung University, Taipei, Taiwan; 6 Graduate Institute of Mind, Brain and Consciousness, Taipei Medical University, New Taipei, Taiwan; 7 Research Center of Sleep Medicine, Taipei Medical University Hospital, Taipei, Taiwan; 8 Department of Electrical Engineering, National Taiwan University, Taipei, Taiwan

**Keywords:** Neuroscience, Cognitive ageing

## Abstract

Preservation of executive function, like inhibition, closely links to the quality of life in senior adults. Although neuroimaging literature has shown enhanced inhibitory function followed by aerobic exercise, current evidence implies inconsistent neuroplasticity patterns along different time durations of exercise. Hence, we conducted a 12-week exercise intervention on 12 young and 14 senior volunteers and repeatedly measured the inhibitory functionality of distinct aspects (facilitation and interference effects) using the numerical Stroop task and functional Magnetic Resonance Imaging. Results showcased improved accuracy and reduced reaction times (RT) after 12-week exercise, attributed to frontoparietal and default mode network effects. In young adults, the first phase (0 to six weeks) exercise increased the activation of the right superior medial frontal gyrus, associated with reduced RT in interference, but in the second intervention phase (six to twelve weeks), the decreased activation of the left superior medial frontal gyrus positively correlated with reduced RT in facilitation. In senior adults, the first six-week intervention led to reduced activations of the inferior frontal gyrus, inferior parietal gyrus, and default mode network regions, associated with the reduced RT in interference. Still, in the second intervention phase, only the visual area exhibited increased activity, associated with reduced RT in interference. Except for the distinctive brain plasticity between the two phases of exercise intervention, the between-group comparison also presented that the old group gained more cognitive benefits within the first six weeks of exercise intervention; however, the cognitive improvements in the young group occurred after six weeks of intervention. Limited by the sample size, these preliminary findings corroborated the benefits of aerobic exercise on the inhibitory functions, implying an age × exercise interaction on the brain plasticity for both facilitation and interference.

## Introduction

### Aging and the advantage of physical exercise

Global aging is an unstoppable trend nowadays. The strategies for approaching healthy aging have evolved into a pivotal concept that attracts international attention, particularly within the field of neuroscience. As individuals age, the cerebral vascular system undergoes a process of deterioration, characterized by the decline in myelin sheaths and neurovascular coupling. These neurophysiological changes have been identified as substantial contributors to the age-related declines in neural processing efficiency, leading to cognitive impairments and poor behavioral performances^1^. These impairments span diverse domains, such as the processing speed of visuospatial abilities and attention, the functionalities of working memory and episodic memory, language production, and executive function^2,3^. Among them, the executive function is particularly relevant to senior adults' quality of life. Its decline potentially leads to an increased frequency of errors in daily activities, rendering it the most vulnerable cognitive function to the aging process^4^. Previous studies have shown that executive function encompassed fundamental subcomponents, such as inhibitory control, task switching, and working memory updating^5^. Notably, among these subcomponents, the function of inhibition has shown the most substantial decline with aging^6^. Physical exercise has been linked to the enhancement of cognitive functioning and has shown as a promising intervention for preserving those three subcomponents of executive function in older adults^7,8^. The underlying neurobiological mechanism through which exercise supports cognitive function has been suggested to involve the enhancement of brain-derived neurotrophic factor (BDNF), which tends to decline with age^9^. Additionally, aerobic exercise not only increases the BDNF concentrations but also elevates the levels of glial fibrillary acidic protein and neuronal nuclei in the brain. This evidence suggested the potential of aerobic exercise to enhance the brain's functionality in the vascular system, thus resulting in an overall improvement in cognitive function^10^. As a result, driven by the benefits of aerobic exercise on the brain, studies within the fields of neuroscience and cognitive psychology have actively delved into investigating the impact of aerobic exercise on the aging brain with the utilization of non-invasive neuroimaging techniques^11,12^.

### Neuroimaging of aging and physical exercise

The intervention of aerobic exercise on executive function in aging individuals has gained recognition for yielding a more pronounced effect when contrasted with other cognitive functions, such as those related to control, spatial, and speed^13^. Nevertheless, how does the brain react with aerobic exercise to attain cognitive improvement? It remains an issue to be investigated because of the sophisticated compensatory mechanisms to preserve cognitive performances^8,14,15^. Considering the distinct regional characteristics of the aging brain, neuroimaging studies provide valuable insights into the potential advantages of exercise in enhancing executive function and even inhibitory control in older adults. Functional magnetic resonance imaging (fMRI) studies have shown that the executive function involves frontal and parietal areas, including the medial prefrontal cortex (mPFC), anterior cingulate cortex (ACC), precentral gyrus, superior frontal, middle frontal, left inferior parietal lobules (IPL), superior parietal lobules (SPL) and the precuneus^16,17^. Along the aging process, a reduction in gray matter volume occurs throughout the whole brain, especially in the frontal area and within the default mode network (DMN)^18,19^. Moreover, age-related reductions in gray matter volume indirectly impact the decreased activation of the right inferior frontal gyrus, pars opercularis, and bilateral IPL during the task about executive function, which is associated with the decline in executive functioning^20^. Additionally, a meta-analysis of fMRI studies has revealed that exercise modulates brain activity in the parietal cortex and DMN area, which consists of the left IPL, SPL, precuneus, and angular gyrus, respectively, in senior adults, whereas in young adults, exercise primarily influences the precuneus and posterior cingulate cortex (PCC), medial regions of DMN^21^. These findings highlight the age-dependent effects of exercise, particularly within the parietal lobe and brain regions associated with DMN. Besides, when focusing on the inhibitory control aspect of executive function, exercise has demonstrated the ability not only to elicit brain activation in the superior frontal gyrus (SFG), superior temporal gyrus (STG), precuneus, and cuneus, but also to reduce activations in the DMN-related regions, precentral area, and limbic system^22^. Collectively, this cumulative evidence supported that exercise interventions influence brain regions associated with the inhibitory control of executive function, encompassing both the frontoparietal networks and the DMN, regardless of age.

### Inconsistent neuroplastic process over time of exercise intervention

Although exercise has been demonstrated to enhance the inhibitory control of executive function, it is important to note that the effect size is relatively small based on findings from the meta-analytic results^8^. Specifically, a 4-week exercise intervention in aging individuals has shown non-significant effects on inhibitory control^23^. Recent research has exhibited that the duration of exercise varied its effects on the parietal lobe^21^. Regarding behavioral outcomes, a recent meta-analytical study highlighted that exercise interventions spanning 1 to 3 months substantially impact executive function among older adults compared to interventions lasting 4 months or more^8^. Altogether, the evidence suggests that exercise-induced neuroplasticity may not be presumed as a linear process, where the neuroplasticity may change the pattern in the middle of consecutive exercise training.

Accordingly, we speculated that such finding might be attributed to the differentiable impact of exercise on distinct cognitive aspects of inhibitory controls (i.e., facilitation and interference), possibly that the tasks within the inhibition group encompass cognitive processes beyond interference alone. For instance, in the Stroop task that has been designed to examine typical inhibitory-control function, the participants exhibited the facilitation effect in the congruent condition (with shorter reaction times and/or higher accuracy than neutral conditions) and the interference effect in the incongruent condition (with longer reaction times and/or lower accuracy)^24–26^. Moreover, although moderate continuous aerobic training has implied the influence of interference effect on the aging brain might enhance efficient cerebral oxygenation^27^, the relationship between the neuroimage-based brain functions and cognitive performances following the different duration of aerobic exercise has not been confirmed. Based on the current scarcity of aging-related studies distinctively investigating the inhibitory controls following physical exercise intervention, the present research aims at unveiling the brain plasticity engaging in the two cognitive processes (facilitation and interference) of the Stroop task at different exercise durations over 12 weeks. In addition, besides targeting the exercise intervention effects on the senior adults, we also recruited young adults for comparison because literature has shown that the aging process alters the neurophysiological responses following exercise intervention^21,28^. Therefore, we hypothesized that the effects of exercise intervention would show age-dependent blood oxygenation level-dependent (BOLD) responses in the DMN and frontoparietal network on facilitation and interference effects, the two distinct cognitive aspects of inhibitory controls, in the Stroop task.

## Materials and methods

### Participants

This study protocol was approved by the Research Ethics Committee of the National Taiwan University Hospital (No. 201805113RINC) and we confirmed that all experiments were performed in accordance with relevant guidelines and regulations. All participants provided their written informed consent to participate in this study.

**Figure 1:**
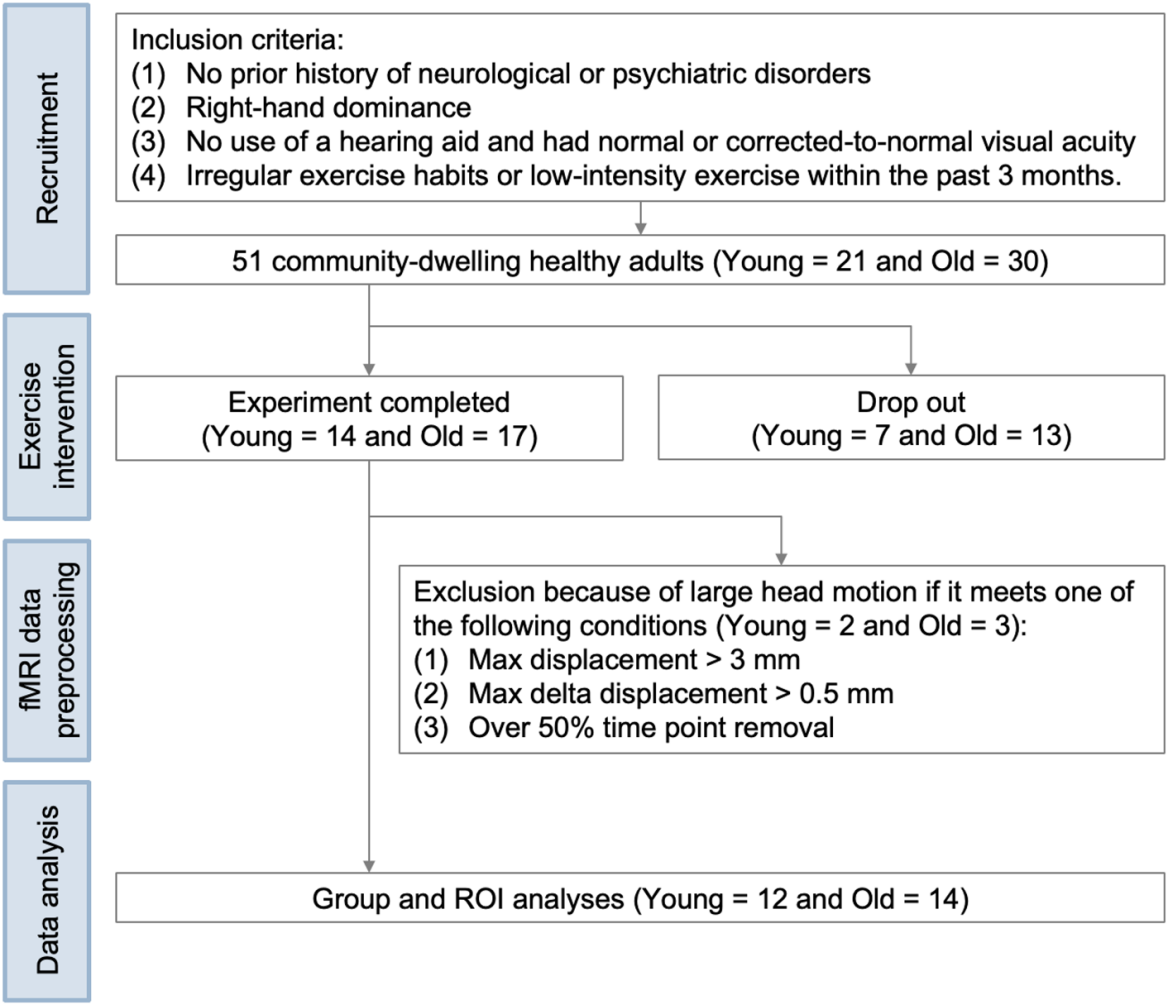
Workflow for the subject exclusion and the fMRI task. Young is the young adult group and Old is the old adult group. ROI is the abbreviation for the region of interest.

A total of 51 community-dwelling healthy young (21 people, age: 23.80 $\pm$ 2.60) and older adults (30 people, age: 64.00 $\pm$ 6.02) participated (all right-handed without regular exercise habits) in this intervention fMRI study. All participants were screened using a detailed self-report health questionnaire with the following inclusion criteria: (1) no prior history of neurological or psychiatric disorders, (2) right-hand dominance, (3) no use of hearing aid and had normal or corrected-to-normal visual acuity, and (4). irregular exercise habits or low-intensity exercise within the past 3 months. Twenty participants were excluded due to drop-out (7 young and 13 old adults), and 5 participants were excluded due to large head motion (Max displacement > 3 mm or Max delta displacement > 0.5 mm, and over 50% time point removal) (2 young and 3 old adults), leading to 26 participants with complete and valid data which were analyzed in the study. These participants were 12 young adults (**Young**, age: 23.20 $\pm$ 0.39) and 14 older adults (**Old**, age: 61.58 $\pm$ 1.27). The workflow detailing participant exclusion during the experiment and analysis processes was illustrated in Fig. 1. There was no significant difference in gender (Fisher’s exact test: Odds ratio = 1.27, *p* = 1.00) and education years (two-sample t-test: *t* = − 1.03,* p* = 0.31) between groups. This information from the two groups was shown in Table 1. To confirm the cognitive performances of the **Old** group to be normal, the Montreal Cognitive Assessment (MoCA) and Mini-Mental State Examination (MMSE) were acquired before the experiment (MoCA: 28.25 $\pm$ 0.45; MMSE: 28.33 $\pm$ 0.37).

**Table 1:** Demographics of voluntary participants in this study.

	Young	Old	Statistic	
**Age**				
Mean (SE)	23.75 (0.65)	61.58 (1.27)	*t value*	-25.30
Range	21–30	52–70	*p value*	< 0.001**
**Gender**				
*Female/male*	7/5	9/5	*p value*	1.00
**Education years**				
* Mean (SE)*	15.50 (0.36)	15.53 (0.49)	*t value*	-1.03
			*p value*	0.31
**Psychological assessment**				
* MoCA*	–	28.25 (0.45)	–	
* MMSE*	–	28.33 (0.37)	–	

SE means stand error. Montreal Cognitive Assessment and Mini-Mental State Examination are abridged to MoCA and MMSE. The age and education years adopt the two-sample t-test to verify the difference between Young and Older. The statistical analysis of gender is calculated using Fisher’s exact test. (*: 0.01 < *p* < 0.05; **: *p* < 0.01).

### Exercise intervention

All participants were asked to conduct a 3-month training of an aerobic exercise program with moderate intensity by spinning bikes (3 times/week, 30 mins/times, BLADEZ H9355i-iSpada 2) at National Taiwan University. The exercise intensity was moderate, and the participants were instructed to maintain the heart rate reserve at 40–59%, predicted based on the age and heart rate of rest. A wireless belt was worn around the chest (BLADEZ, Polar T34), monitoring the heart rate during exercise. The spinning bike automatically adjusted the pedaling resistance according to the real-time heart rate of each participant. This training time was fixed from 4:00 pm to 7:00 pm to avoid confounding circadian variations.

### MRI data acquisition

The MRI experiment was conducted by a 3-Tesla MRI scanner (SIEMENS MAGNETOM Prisma) with a 20 channels head coil at National Taiwan University. The time points of MRI data acquisition included three-time of Week-0, Week-6, and Week-12 exercises, in total. Brain anatomy was acquired using a T_1_-weighted image with 3D magnetization-prepared rapid gradient echo (MPRAGE) sequence (matrix size: 256×256×192; resolution: 0.93×0.93×0.93 mm^3^; inversion time = 900 ms; repetition time (TR) = 2000 ms, echo time (TE)= 2.3 ms; flip angle (FA)= 8°; bandwidth= 200 Hz/pixel; NEX= 1), and the acquisition time was 4 min 40 sec. The functional images were acquired using a single-shot gradient-echo echo-planar imaging (GE-EPI) sequence (matrix size: 64×64×31; thickness=3.5 mm; field of view: 224×224 mm2; TR = 2000 ms, TE= 32 ms; FA= 90°; bandwidth= 3005 Hz/pixel).

### Functional MRI tasks and procedure

The modified version of the physical-numerical interference paradigm (i.e., numerical Stroop task) was utilized for functional MRI study^26,29^. These experimental procedures of the numerical Stroop task for each run had the sample stimuli for the congruent and incongruent conditions, which each had two blocks in each run, and the congruent and incongruent conditions represented facilitation and interference effects, respectively. Each trial comprised a blank screen lasting 1000 ms, which was subsequently followed by a stimulus presentation lasting 1000 ms, and there were 18 trials per block. Participants would judge numerical magnitude in this task when the screen presented a pair of digits. They were asked to respond which was numerically larger while ignoring their physical size. The congruent condition meant the congruence between the numerical magnitude and physical size, and the incongruent condition meant the opposite. The time from a pair of digits that occurred to the response of the participants is referred to as the reaction time (RT). The accuracy of the numerical Stroop task referred to the percentage of correct responses by participants for each condition. The acquisition time of each numerical Stroop task was 5.4 min, and every participant was asked to conduct the task twice.

### Preprocessing and image analysis

**Figure 2:**
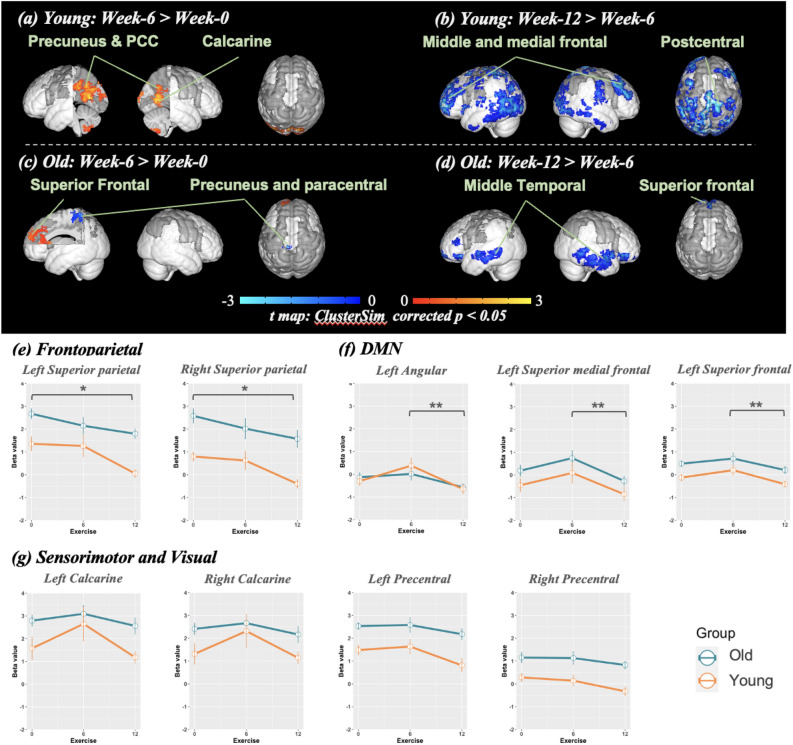
The brain-activity differences of the congruent condition in the numerical Stroop task across the two phases of exercise intervention. (**a**-**d**) the spatial pattern of brain activities of both Young and Old groups. Multiple comparison correction of these activation maps adoptes the ClusterSim corrected to p < 0.05. (**e**–**g**) the changes of fMRI signals across the three times points, where the regions were prescribed from AAL3, encompassing frontoparietal, default mode network (DMN), and sensorimotor and visual areas. Green and orange lines indicate Old and Young groups, respectively. The "*" indicates the significance level 0.01 < *p* < 0.05, and "**" means the *p* < 0.01 with the Bonferroni test.

**Figure 3:**
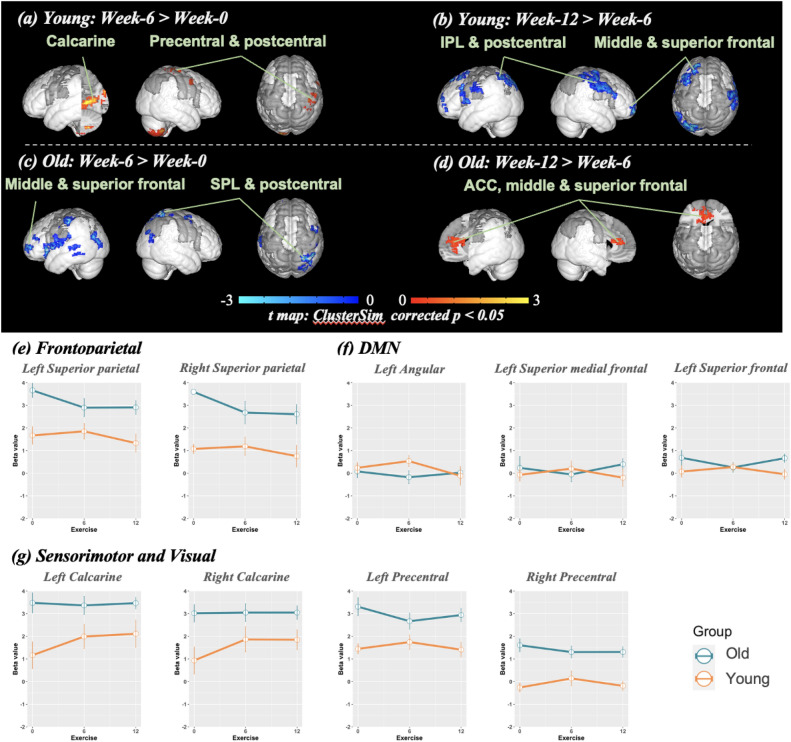
The brain-activity differences of the incongruent condition in the numerical Stroop task across the two phases of exercise intervention. (**a**-**d**) the spatial pattern of brain activities of both Young and Old groups. Multiple comparison correction of these activation maps adoptes the ClusterSim corrected to p < 0.05. (**e**–**g**) the changes of fMRI signals across the three times points, where the regions were prescribed from AAL3, encompassing frontoparietal, default mode network (DMN), and sensorimotor and visual areas. Green and orange lines indicate Old and Young groups, respectively. The "*" indicates the significance level 0.01 < *p* < 0.05, and "**" means the *p* < 0.01 with the Bonferroni test.

The fMRI image preprocessing by Analysis of Functional Neuro Images (AFNI) (Cox, 1996). was as below: (1) Motion correction, (2) Slice timing, (3) Co-register with T1-weighted image, (4) Smoothing, and (5) Normalize to the MNI space. Additionally, in the numerical Stroop task, the time points of large motion would be removed if they meet two criteria: displacement > 3 mm and framewise displacement > 0.5 mm. If the data had over 50% time point removal, this data would be excluded. The activations of the numerical Stroop task were calculated by general linear model (GLM) analysis and averaged beta weights of the two Stroop sessions. For those participants unable to complete the two times of numerical Stroop tasks or presented big head movement during one scanning, only one task scan was included as the numerical Stroop profile (Supplementary result 1). The AFNI-3dMVM was adopted for the multivariate analysis, where the gender was treated as the covariate, as shown in Figs. 2a–d and 3 a–d.

### Statistical analysis

In the region of interest (ROI) analysis, we used the Automated Anatomical Labelling Atlas (AAL3) template to select 22 areas, including DAN and DMN task-induced by the numerical Stroop task and sensorimotor and visual areas (Supplementary result 2 and 3). The R (Version 3.6.3) and R studio (Version 1.1.463) for statistical analysis processed two-way Analysis of Variance (ANOVA, Age × Exercise) and correlation analysis. A two-way ANOVA was applied to observed exercise intervention and the aging effect, and the post-hoc analysis was adopted with Bonferroni correction. Moreover, the Pearson correlation analysis was used to estimate the relations between the fMRI signals and the RTs at the different timings of the exercise program. *p* < 0.05 was considered significant.

### Ethics approval

The study protocol was approved by the Research Ethics Committee of the National Taiwan University Hospital (No. 201805113RINC). All experiments were performed in accordance with relevant named guidelines and regulations, and all participants provided their written informed consent to participate in this study.

## Results

### The effects of exercise intervention on cognitive performance

Table 2 shows the average performances of the numerical Stroop task, including both RT and accuracy of the Young and Old groups over the three states (Week-0, Week-6, and Week-12). For both congruent and incongruent conditions, Age and the Exercise showed significant main effects in both RT and accuracy; however, they did not exhibit significant interaction (RT in Congruent: *F*_*2,48*_ = 1.53, *p* = 0.23, RT in Incongruent: *F*_*2,48*_ = 0.47, *p* = 0.63, accuracy in Congruent: *F*_*2,48*_ = 0.47, *p* = 0.63, accuracy in Incongruent: *F*_*2,48*_ = 0.47, *p* = 0.63). The RT of the Old group was longer than the RT of the Young group (Congruent: *F*_*1,24*_ = 8.96, *p* < 0.006, Incongruent: *F*_*1,24*_ = 10.53, *p* < 0.003), and the accuracy of the Old was lower than one of the Young (Congruent: *F*_*1,24*_ = 10.53, *p* < 0.003, Incongruent: *F*_*1,24*_ = 10.53, *p* < 0.003). After the exercise, the RT was reduced (Congruent: *F*_*2,48*_ = 12.79, *p* < 0.001, Incongruent: *F*_*2,48*_ = 17.89, *p* < 0.001), and the accuracy increased (Congruent: *F*_*2,48*_ = 17.89, *p* < 0.001, Incongruent: *F*_*2,48*_ = 17.89, *p* < 0.001).

**Table 2:** The descriptive statistics of behavioral indices.

*Performance*	Reaction time (SE)		Accuracy (SE)			
	*Young*	*Old*	*Young*	*Old*		
**Congruent**						
*Week-0*	569.36 (21.02)	614.85 (14.77)	0.98 (0.00)	0.94 (0.02)		
*Week-6*	538.46 (21.76)	614.18 (14.38)	0.98 (0.01)	0.97 (0.01)		
*Week-12*	510.13 (18.33)	586.83 (14.20)	0.99 (0.00)	0.98 (0.01)		
**Incongruent**						
*Week-0*	638.74 (24.33)	699.42 (14.01)	0.95 (0.01)	0.93 (0.02)		
*Week-6*	611.64 (20.60)	690.79 (11.28)	0.96 (0.01)	0.93 (0.02)		
*Week-12*	588.57 (20.20)	660.70 (13.40)	0.96 (0.01)	0.95 (0.01)		

*2-way ANOVA*	*Age group*	*Exercise*	*Group:Exercise*	*Age group*	*Exercise*	*Group:Exercise*
**Congruent**						
*F value*	8.96	12.79	1.53	10.53	17.89	0.47
*p value*	0.006**	< 0.001**	0.23	0.003**	< 0.001**	0.63
*ges*	0.23	0.09	0.01	0.27	0.11	0.00
**Incongruent**						
*F value*	10.53	17.89	0.47	10.53	17.89	0.47
*p value*	0.003**	< 0.001**	0.63	0.003**	< 0.001**	0.63
*ges*	0.27	0.11	0.00	0.27	0.11	0.00

This table records the reaction time and accuracy during the numerical Stroop task, which includes the congruent and incongruent conditions, and the two-way analysis of variance (ANOVA) verifies statistical significance for age group (Age), exercise periods (Exercise), and their interaction (Group: Exercise). (*: 0.01 < p < 0.05; **: p < 0.01).

### The effects of exercise intervention on the Young and the Old brain

The brain changes after the exercise intervention were illustrated in Fig. 2 for the congruent condition and Fig. 3 for the incongruent condition, which presented the effects of the exercise intervention on functional brain activation across three-time points of exercise intervention periods in congruent and incongruent conditions. The detailed locations of brain activities were listed in Supplementary result 4. In the congruent condition, we found that both age groups showed similar trends, but the exercise effect of the first phase (within 6 weeks) was different from that of the second phase (from Week-6 to Week-12). After 6 weeks of exercise, the brain activity of the congruent condition was increased in the left precuneus, cuneus, angular gyrus, calcarine, and right cerebellum in the Young adults (Fig. 2a), but the contrast between Week-0 and Week-6 of the Old group involved an increment in the SFG and a decrement in the precuneus and paracentral lobule (Fig. 2c). The brain changes of the congruent condition in the late phase (Week-12 vs. Week-6) showed a reduction in both groups. The Young demonstrated the lower activity in the middle temporal gyrus and postcentral gyrus, hippocampus and inferior temporal gyrus, and middle frontal gyrus (Fig. 2b), while the Old group showed reduced activity in the middle temporal gyrus, which cluster including the SFG (Fig. 2d). The ROI analysis of DAN, DMN, sensorimotor, and visual cortex showed the age and exercise effect but no significant interaction between age and exercise. The post hoc test depicted the changes in the bilateral superior parietal lobe (SPL), a portion of DAN, between Week-0 and Week-12, shown in Fig. 2e (Left: *t*_*25*_ = − 1.98, *p* < 0.011; Right: *t*_*25*_ = − 2.11, *p* < 0.033). The left angular gyrus, left SFG, and left superior medial frontal cortex of DMN showed differences between Week-6 and Week-12 (Angular: *t*_*25*_ = − 0.62, *p* < 0.005; superior frontal: *t*_*25*_ = 0.36, *p* < 0.021; superior medial frontal: *t*_*25*_ = 0.20, *p* < 0.010), which results were displayed in Fig. 2f. Conversely, the sensorimotor and visual showed insignificance change in exercise periods (Fig. 2g).

Additionally, exercise intervention presented opposite trends in brain changes between Young and Old during the execution of the incongruent condition. For the incongruent condition patterns between Week-6 and Week-0, the Young presented positive changes in the left lingual gyrus and calcarine, and the right postcentral, precentral, putamen, caudate, and cerebellum (Fig. 3a). In contrast to the incongruent condition patterns between Week-6 and Week-0, the Old group had negative trends in the left inferior frontal, right superior parietal lobule, and bilateral middle frontal, superior frontal, postcentral, and middle temporal gyrus (Fig. 3c). The brain patterns of bilateral postcentral and middle frontal gyrus, left superior and inferior frontal, middle occipital gyrus, cerebellum, and right middle orbital gyrus were influenced in the second phase (Week-6 vs. Week-12) in the Young group during incongruency (Fig. 3b). Conversely, the Old involved positive changes in the left anterior cingulate cortex (Fig. 3d). For ROI analysis, the 2-way ANOVA results of the incongruent condition revealed no interaction between age group and exercise. This finding only had the main effect of age group, which significantly occurred in DAN, DMN, sensorimotor, and visual cortex (Fig. 3e–g). The 2-way ANOVA and post hoc results in 22 ROIs were placed in Supplementary result 5 and 6, along with the individual trajectory across the three measurements.

### The association between brain activation and cognitive performance

**Figure 4:**
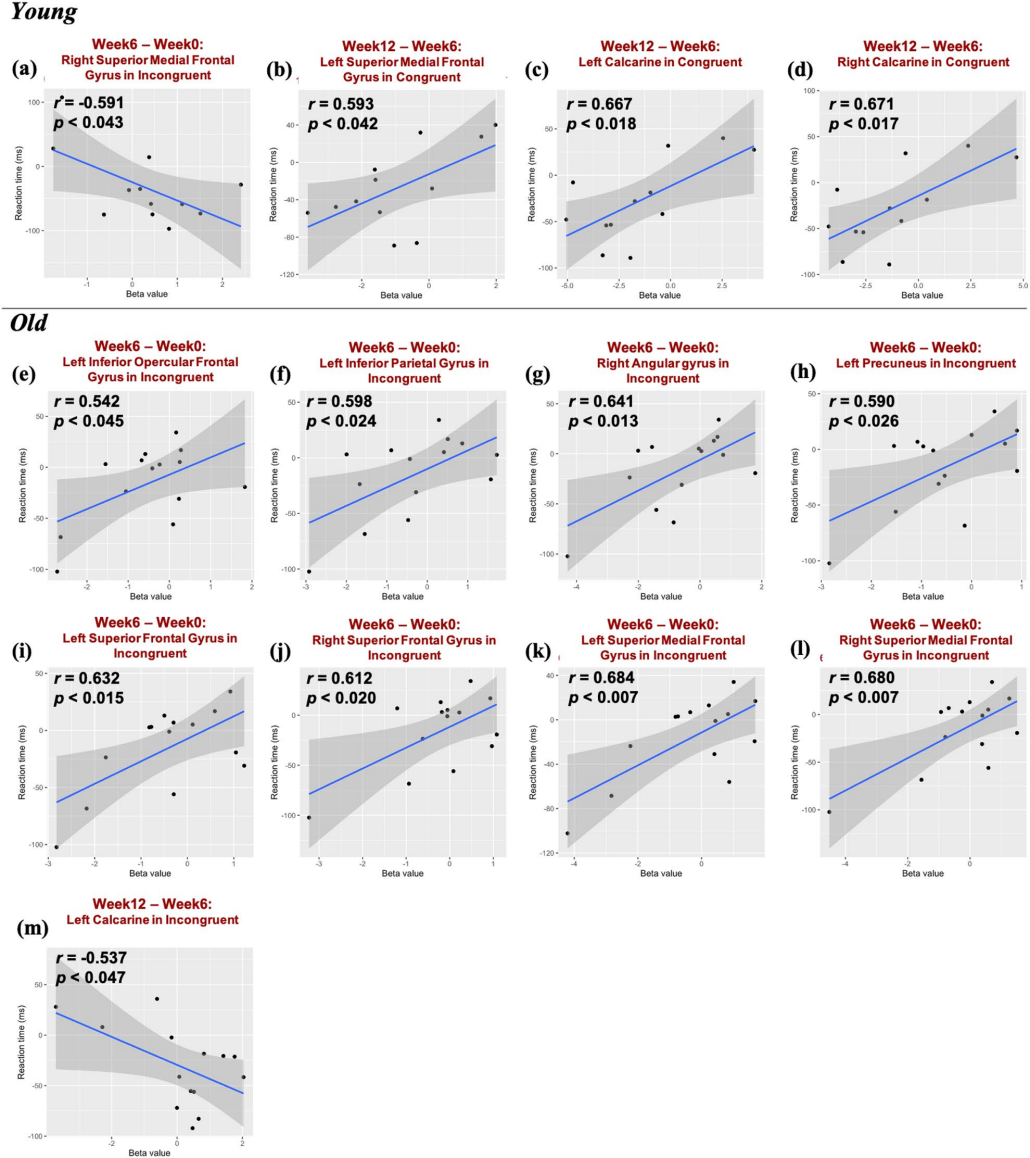
Pearson’s correlation between the brain-activity changes and the RT changes in the congruent condition of the numerical Stroop task for both Young and Old groups. "r" indicates Pearson’s correlation coefficient, and "p" stands for the *p*-value.

The investigation into the brain changes of exercise effect adopted Pearson's correlation with RT. During the incongruent condition of Young, significant correlations were found between fMRI signals of the right superior medial frontal and changes in RT (r = 0.59, p < 0.043) in the first phase (Week-6 vs. Week-0), as shown in Fig. 4a. In the second phase (Week-12 vs. Week-6), the correlation between fMRI signals of the left superior medial frontal gyrus and the bilateral calcarine and the changes in RT (Left superior medial frontal: r = 0.59, p < 0.042, Left calcarine: r = 0.68, p < 0.018, Right calcarine: r = 0.67, p < 0.017) was significant during the congruent condition of Young, as shown in Fig. 4b-d. Moreover, the difference in fMRI signals of the left inferior opercular frontal gyrus, left inferior parietal lobe, right angular gyrus, left precuneus, left superior frontal gyrus, right superior frontal gyrus, left superior medial frontal gyrus, and right superior medial frontal gyrus in Old during the incongruent condition for different exercise periods was a significant correlation in RT changes (Left inferior opercular frontal gyrus: r = 0.54, p < 0.045; Left inferior parietal lobe: r = 0.60, p < 0.024; Right angular gyrus: r = 0.64, p < 0.013; Left precuneus: r = 0.59, p < 0.026; Left superior frontal gyrus: r = 0.63, p < 0.015; Right superior frontal gyrus: r = 0.61, p < 0.020; Left superior medial frontal gyrus: r = 0.68, p < 0.007; Right superior medial frontal gyrus: r = 0.68, p < 0.007) in the first phase (Week-6 vs. Week-0), as shown in Fig. 4e–l. In the second phase (Week-12 vs. Week-6), the correlation between fMRI signals of the left calcarine and the changes in RT was a significantly negative correlation during the incongruent condition (r = − 0.54, p < 0.047), as shown in Fig. 4m. On the other hand, during the congruent condition, the fMRI signals in 22 ROIs indicated no significant correlation with RT changes between exercise periods in Old.

## Discussion

The current work provides three new aspects to the fields of aging and exercise intervention: (1) exploring the benefits of exercise intervention for both young and older adults in cognitive aging, (2) utilizing neuroimaging to measure the brain plasticity at three different timings along the exercise intervention, and (3) examining the impact of exercise intervention distinctively on facilitation (Congruent condition) and inhibition (Incongruent condition) of inhibitory function. Specifically, we measured the 12-week exercise effect of different age groups (Young vs. Old) on the Stroop facilitation (congruent condition) and interference (incongruent condition) processes and its neurocognitive mechanisms. Our findings indicated three major points in terms of the facilitation performance of the numerical Stroop task: (1) Age-related changes in functional plasticity in brain activity and cognitive-control performances; (2) both brain and behavioral indices showed distinctive effects between the first phase (Week-6 vs. Week-0) and the second phase (Week-12 vs. Week-6) of the exercise intervention; and (3) brain-behavioral associations showed the reduced brain activity following exercise intervention, especially in the frontoparietal and DMN regions, associated with fast response speed in facilitatory and inhibitory control in young adults but inhibitory controls in old adults.

The exercise intervention resulted in faster RT in the numerical Stroop task. However, the Young and Old had distinct brain activity changes. The positive impact of exercise intervention on the Young focused on the second phase of facilitation responses, enhancing RT through decreased activation in the left superior medial frontal gyrus of the DMN and bilateral calcarine (Fig. 4b–d). On the other hand, the Old showed improved RT in the interference process, correlating with decreased activation in the left frontoparietal region and bilateral DMN during the first phase of exercise intervention (Fig. 4e–l). The study also identified that increased activation in the right superior medial frontal gyrus for the Young during the first phase and in the left calcarine for the Old during the second phase of exercise intervention might contribute to enhanced interference responses (Fig. 4a and m).

This study contributes to the aging and exercise intervention field in three aspects: First, the neurocognitive benefits of exercise intervention were identified both in young and older adults, providing supportive evidence for preventing age-related declines in cognitive controls. Second, functional brain plasticity was observed at three-time points (Week-0, Week-6, and Week-12 exercises) of exercise intervention. Finally, different aspects of cognitive-control function were simultaneously examined during exercise intervention, with inhibitory and facilitatory controls showing age-sensitive effects after intervention. In brief, this study may imply an Age × Exercise interaction on the brain plasticity for improving both facilitation and interference of the inhibitory function. Nevertheless, due to the small sample size, caution shall be exercised in interpreting the results.

### Effect of the exercise intervention on facilitation and interference

After a 12-week aerobic exercise intervention, behavioral improvements were observed in both facilitation and interference performance, with faster and more accurate responses. The brain activation associated with cognitive control processes varied across different exercise periods, as evidenced by beta values extracted from ROIs. Specifically, the BOLD responses demonstrated a significant influence for exercise intervention on facilitation processes and non-significance on interference processes. In the facilitation process, the activations of the frontoparietal (bilateral SPL) decreased in the first phase and the DMN (left angular gyrus, superior medial frontal, and superior frontal) reduced in the second phase of the exercise intervention. The SPL was considered a part of regulating visuospatial attention and played a role in the numerical process during the numerical Stroop task^30,31^. Additionally, the task-induced DMN deactivation has been observed the decline following the aging process, which might be associated with a functional deficit in the cognitive control and resource allocation^32,33^. The aerobic exercise contributed to a stronger DMN deactivation, thereby aiding in a higher efficient regulation of attentional resources during task execution. The long-term exercise intervention could increase the concentration of BDNF, whose upregulation can improve neurogenesis and synaptogenesis and mitigate neuronal loss, thereby increasing neural efficiency to influence specific brain regions^34–38^. Furthermore, the reduced DMN activity may reflect lower emotion arousal and better emotion regulation, promoting executive function^39^. Therefore, the improved facilitation process after 12 weeks of exercise intervention could originate from the enhancement of numerical judgment and cognitive-emotion interactions, further promoting behavioral performance.

In addition, the RT of the interference process decreased significantly following the exercise periods. While the impact of 12-week exercise intervention on brain activity can be observed, this alteration associated with enhancing RT appears to be more pronounced in older individuals. This might explain why the results of the 2-way ANOVA did not reach statistical significance in the interference process. The activation map during the interference process in the numerical Stroop task was similar to the one during the facilitation, and the interference could make stronger activation in the frontoparietal lobe than facilitation^29,30^. Regarding the interference process, previous studies considered the regions of interference control focused on the dorsolateral prefrontal cortex (DLPFC), where the inferior frontal and middle frontal gyri especially involved conflict resolution in the numerical Stroop task^40,41^. While short-period exercise has been found to increase cerebral oxygenation in the frontal lobe, it has yet to be verified whether cerebral oxygenation positively impacts cognitive performance following long-term exercise^42,43^. Such changes, to be reflected in the relationship between brain activity and RT, might require longer exercise interventions or more sample size.

### Neuroimaging changes supporting cognitive enhancement in young and old

The behavioral improvement in the Young from Week-6 to Week-12 was associated with a decrease in bilateral calcarine activation. The calcarine region is often involved in the initial sensory processing of visual features in higher-order cognitive tasks^44^. Moreover, it plays an important role in feature discrimination^45,46^. On the other hand, in Young, activation in the superior medial frontal gyrus of the DMN exhibited an increasing trend from Week-0 to Week-6, followed by a decrease from Week-6 to Week-12. These changes in brain imaging were correlated with the RT in the interference and facilitation processes, respectively. While the implications of negative activation are not fully elucidated, past research suggests a negative correlation between the intensity of DMN activation in the frontal lobe and the neurotransmitter GABA associated with neural inhibition^47^. Therefore, the early imaging changes during exercise might arise from increased activity in task-related brain regions due to improved cardiorespiratory function and increased cerebral blood flow^48,49^. The second phase exercise might elevate the BDNF concentration^50^, exerting neuroprotective effects and enhancing neural function^51^. From the perspective of exercise in preventing aging, the benefits of exercise for the Young might stem from increased cerebral blood flow and BDNF, thereby achieving neuroprotection and delaying aging^52^. However, the assistance from six weeks of exercise appears more pronounced for the interference process, while the benefits for the facilitation process may take up to twelve weeks to manifest.

We found age-related decreased brain activations in the left inferior frontal gyrus and IPL was associated with improved interference response after 6-week exercise. Previous studies have suggested that older adults tended to exhibit a shift towards bilateral brain activation to promote cognitive performances compared to young adults who predominantly utilize unilateral brain resources to execute cognitive tasks^14,29,53^. However, our study provides another speculation suggesting that Aerobic exercise intervention could facilitate the neural function of older individuals and promote a more youthful process of brain functionality. After 6 weeks of exercise intervention in Old, the DMN regions modulated the improvement in RT of interference. The potential factors contributing to these findings might stem from the sensitivity of the PCC, the core of DMN, to changes in oxygen levels and its association with cerebral blood supply and storage^54,55^. A previous study also found that cerebrovascular reactivity in DMN increasing was associated with the enhancement of cardiorespiratory fitness after exercise^56^. Exercise intervention might enhance behavioral performance in older individuals by modifying the efficiency of cerebral blood supply in the posterior brain region. Additionally, we also observed a correlation between the increased imaging results in the calcarine region and improved interference performance in the Old from Week-6 to Week-12 of exercise intervention. Previous fMRI visual experiments have indicated that the BOLD signal in the occipital lobe of older adults is lower than in young adults^57^. Additionally, rodent studies have demonstrated that exercise intervention can enhance neuroplasticity in primary visual cortex adult mice^58^. Therefore, these changes in the visual area of older adults due to exercise intervention might contribute to a brain pattern that aligns them more closely with younger individuals.

Furthermore, the distinctive time periods of exercise intervention (Week-6, Week-12) worked on different brain regions, which might be stemmed from the diverse sequence of exercise-induced plasticity in the brain. The potential mechanisms underlying the enhancement of brain plasticity by exercise may involve increases in local neurotrophic factors, improvements in vascular supply efficiency, and alterations in gray and white matter volumes^56,59–61^. Additionally, prior research has reported an augmentation in gray matter volume within the frontal, parietal, and cingulate regions following a 6-month exercise intervention and has implied the impact of varying exercise durations on different brain regions exhibits inconsistent variation curves, which partially support our findings in terms of longitudinal neuroplasticity^62,63^. In studies of physical exercise, there has been a growing recognition of the potential neuroplasticity in the hippocampus in recent years^64^. In addition to focusing on the numerical Stroop task, concerning the potential effect of exercise intervention on memory-related hippocampus, we also conducted additional analyses of the hippocampus regions (AAL3) for both groups, presented in Supplementary result 7.

### Negative BOLD of DMN in exercise effect

Based on our findings, this negative activation was observed in the DMN baseline for both the Young and Old groups. However, the magnitude of such deactivation was greater in the Young group, as compared to the Old group, and the deactivation of the Old group was more prominent in the posterior DMN (PCC and angular gyrus). During high cognitive demand tasks, negative activation could be observed in the regions of the DMN^65^. While the precise mechanisms underlying the negative activation of the DMN are not fully understood, previous findings indicate that younger individuals exhibit a greater degree of negative activation than older individuals^66^, in agreement with our observations. Furthermore, a reduced spatial extent of negative DMN activation may be considered as an indicator of cognitive decline^67^. Therefore, we verified that the 12-week aerobic exercise intervention could facilitate cognitive performance through the level of negative BOLD in the DMN, especially in the elderly.

### Limitations

First, our sample size was small, lower than 20 per group. Nevertheless, the results could be supported by similar neuroimaging outcomes of the frontoparietal region and DMN during the numerical Stroop task. Previously, one review study reported the sample size of previous studies for a long-term exercise intervention in different age groups was usually small, which implied difficulty in recruitment for long-term interventional studies on different age groups, particularly older adults^68^. Regarding the past longitudinal studies with exercise intervention, especially those involving aerobic exercise with spinning bikes in older adults, the sample size typically ranged from 8 to 17 individuals^50,69–71^. In our design, we initially recruited 30 senior adults, but the drop rate reached 43% due to the 3-time fMRI measurements with a 3-month exercise intervention, which was the biggest challenge in this study. Therefore, to check the achieved statistical power of our findings, we adopted post hoc testing (G power 3.1) over the ROI-based 2-way ANOVA model. Among the 22 ROIs in the congruent condition, most of the brain regions achieved power of over 74.7%; only 4 brain regions showed power of less than 29.7% (bilateral precuneus, left superior medial frontal gyrus, and right postcentral gyrus). Similarly, in the incongruent condition, most of the brain regions achieved power of over 94.7%, while 6 brain regions showed power of less than 43.2% (right PCC, left angular gyrus, bilateral precuneus, left superior medial frontal gyrus, and right postcentral gyrus). Even though there is still a lack of confidence to generalize the findings based on the shortage of sample size. However, our preliminary results delve into potential impacts that should be noticed. Moreover, they may offer valuable insights for future meta-analyses in this field. Secondly, our project only observed 12 weeks of aerobic exercise intervention because the confirmation of this exercise intervention could be better than over 12 weeks in the improvement of executive function^8^. Although the observation of the effect on the interference process was not significant on the ROI analysis after 12 weeks of exercise, we still present the trend of brain-activity changes in the interference process after a longer duration of exercise intervention, which warrants further investigation in the near future. Third, although the accuracy could serve as a behavioral measure for facilitation and interference effects, all participants in our study maintained an accuracy rate of over 95%, with the young group reaching 98% in congruent condition accuracy at Week 0 and increasing to 99% after exercise intervention (Table 2). Using accuracy to explore correlations between pre- and post-intervention changes and imaging might obscure significant variations due to its ceiling effect. For these reasons, we only employed the reaction time as the behavioral indicator for facilitation and interference effects in this study.

## Conclusions

This MRI study revealed that a 12-week aerobic exercise intervention enhanced performance in a numerical Stroop task and also confirmed that while young and older adults showed performance improvements following a 12-week exercise intervention, these brain changes might be shifted nonlinearly over the 12-week exercise intervention. In young adults, through improved efficiency in visual sensory processing and DMN regions, the RT of the numerical Stroop task could be enhanced. Still, the assistance from 6 weeks of exercise appears more pronounced for the interference process, while the benefits for the facilitation process might take up to 12 weeks to manifest. On the other hand, older adults reallocated their brain resources in regions associated with numerical processing and judgment, shifting closer to the functional pattern observed in young adults. The exercise intervention further enhanced the efficiency of blood supply in the posterior brain, resulting in improved interference performance.

### Supplementary Information

Supplementary Information.

## Data Availability

The datasets generated during and/or analyzed during the current study are available from the corresponding author upon reasonable request.
