# Supplementary material for: Functional neuroplasticity of facilitation and interference effects on inhibitory control following 3-month physical exercise in aging
Source: Sci Rep. 2024 Feb 14;14:3682. doi: 10.1038/s41598-024-53974-5 (PMC10866924; doi:10.1038/s41598-024-53974-5)
Supplement: Supplementary file 1 — Supplementary Information. [file 41598_2024_53974_MOESM1_ESM.pdf]

Supplementary result 1. Data type included in Stroop analysis for three longitudinal scans.

	Number		
	Young	Old	Young + Old
**Stroop*1**	12	8	20
**Stroop*2 to average**	24	34	58
**Total**	36	42	78

This proportion of participants with completed Stroop task two times was 74.36 %, and the proportion of the single-time scan was 25.64 %.

Supplementary result 2. Region of interest from AAL3

Networks	AAL3 template	Brain regions
Dorsal attention network	Frontal_Inf_Oper_L	Left inferior frontal gyrus, opercular part
	Frontal_Inf_Oper_R	Right inferior frontal gyrus, opercular part
	Parietal_Inf_L	Left inferior parietal gyrus, excluding supramarginal and angular gyri
	Parietal_Inf_R	right inferior parietal gyrus, excluding supramarginal and angular gyri
	Parietal_Sup_L	Left superior parietal gyrus
	Parietal_Sup_R	Right superior parietal gyrus
Default mode network	Cingulate_Post_L	Left posterior cingulate gyrus
	Cingulate_Post_R	Right posterior cingulate gyrus
	Angular_L	Left angular gyrus
	Angular_R	Right angular gyrus
	Precuneus_L	Left precuneus
	Precuneus_R	Right precuneus
	Frontal_Sup_L	Left superior frontal gyrus, dorsolateral
	Frontal_Sup_R	Right superior frontal gyrus, dorsolateral
	Frontal_Sup_Med_L	Left superior frontal gyrus, medial
	Frontal_Sup_Med_R	Right superior frontal gyrus, medial
Sensorimotor and visual	Postcentral_L	Left postcentral gyrus
	Postcentral_R	Right postcentral gyrus
	Precentral_L	Left precentral gyrus
	Precentral_R	Right precentral gyrus
	Calcarine_L	Left calcarine fissure and surrounding cortex
	Calcarine_R	Right calcarine fissure and surrounding cortex

Supplementary result 3. Region of interest from AAL3 in visual presentation

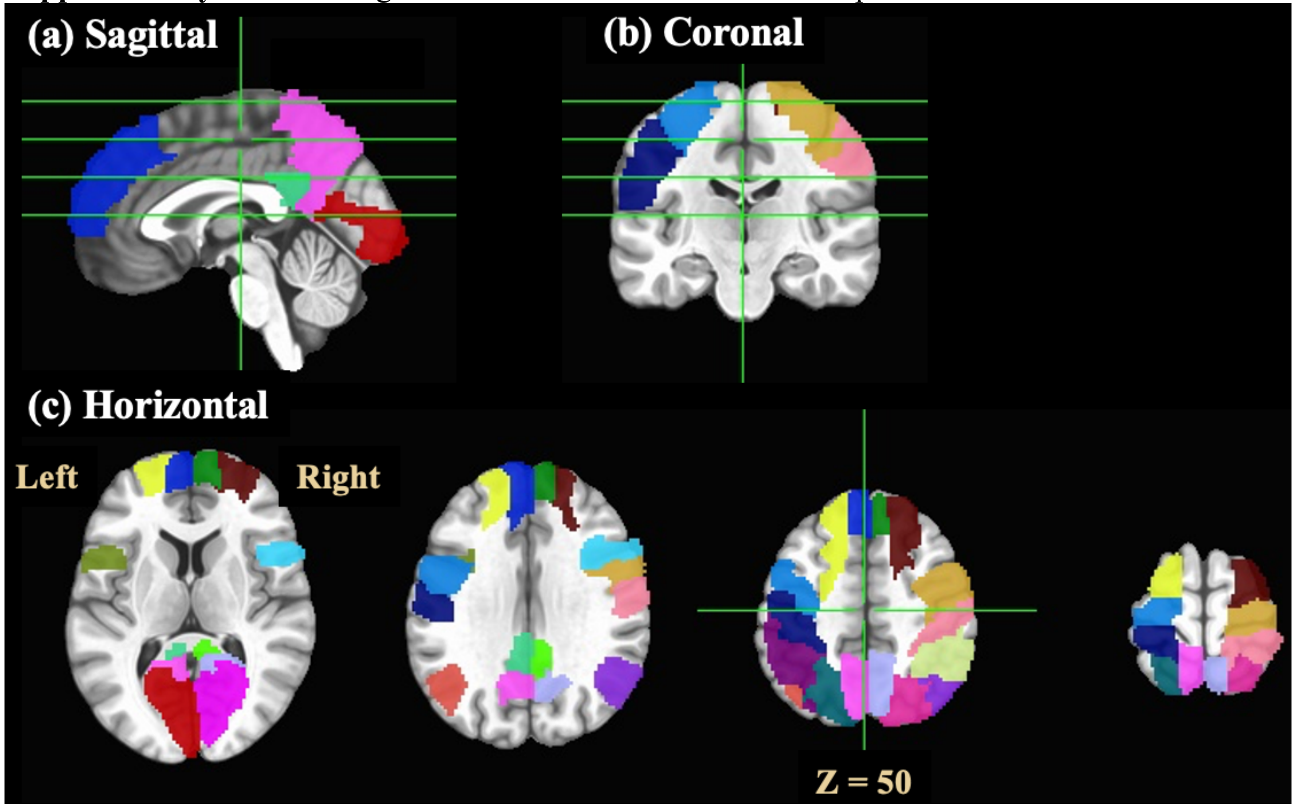

Supplementary result 4. Brain-activity difference in the numerical Stroop task across the two phases of exercise intervention.

Brain region	Hemisphere	Voxels	CM x	CM y	CM z	Mean	SEM	Max Int	Peak x	Peak y	Peak z
**Congruent**											
Old group: Week-6 > Week-0											
Superior Frontal Gyrus	L	1378	7.2	-36.0	25.5	0.95	0.01	2.61	8	-68	26
Precuneus and Paracentral Lobule	L	598	15.2	41.6	63.1	0.92	0.02	-4.34	6	32	80
Old group: Week-12 > Week6											
Middle Temporal Gyrus	B	10223	-2.6	-16.2	2.7	1.17	0.01	-4.63	-4	-72	8
Young group: Week-6 > Week-0											
Precuneus and Cuneus	L	3614	0.6	66.8	23.8	1.83	0.01	4.66	-2	62	36
Cerebellum	R	914	-17.2	63.6	-53.2	1.06	0.01	2.58	4	68	-52
Angular Gyrus	L	757	44.4	72.0	33.7	1.44	0.02	3.56	34	84	46
Young group: Week-12 > Week6											
Middle Temporal Gyrus and Postcentral Gyrus	L	30460	12.1	41.5	25.4	1.47	0.00	-5.99	4	74	58
Hippocampus and Inferior Temporal Gyrus	R	1147	-27.1	25.0	-7.7	1.26	0.02	-3.86	-24	22	-10
Middle Frontal Gyrus	R	888	-43.9	-28.1	37.1	1.54	0.02	-3.17	-24	-32	60
**Incongruent**											
Old group: Week-6 > Week-0											
Superior Parietal Lobule and Postcentral Gyrus	R	1220	-31.3	42.4	55.6	1.22	0.02	-5.71	-34	56	68
Middle Frontal Gyrus and Superior Frontal Gyrus	L	1055	25.2	-45.9	2.7	1.09	0.01	-2.84	44	-58	2
Postcentral Gyrus	L	812	55.8	12.5	22.0	1.26	0.02	-3.75	64	22	46
Middle Temporal Gyrus	L	567	47.9	58.7	15.1	1.07	0.02	-2.83	58	66	20
Inferior Frontal Gyrus	L	545	43.0	-20.2	-0.4	1.44	0.03	-3.76	34	-6	-18
Middle Temporal Gyrus	R	538	-43.7	65.7	16.5	0.94	0.01	-2.06	-52	74	30
Middle Frontal Gyrus and Superior Frontal Gyrus	R	464	-28.2	-46.2	17.8	0.81	0.01	-1.73	-28	-56	12

Old group: Week-12 > Week6											
Anterior Cingulate Cortex	L	934	6.5	-37.2	12.6	0.84	0.01	1.77	6	-52	10
Young group: Week-6 > Week-0											
Lingual Gyrus and Calcarine Gyrus	L	1073	17.7	70.0	3.1	1.87	0.02	3.83	18	62	4
Cerebellum	R	670	-11.6	55.9	-51.9	1.45	0.04	4.29	2	48	-50
Postcentral and Precentral Gyrus	R	649	-40.0	22.2	51.8	0.96	0.01	2.36	-52	-4	46
Putamen and Caudate	R	546	-14.2	-22.8	-7.1	1.59	0.02	2.79	-8	-16	-8
Young group: Week-12 > Week6											
Middle Frontal Gyrus and Superior Frontal Gyrus	L	990	24.5	-26.0	50.7	1.27	0.02	-4.29	24	-44	50
Postcentral Gyrus	R	842	-56.6	13.5	42.3	1.34	0.01	-3.44	-50	14	62
Postcentral Gyrus and Inferior Frontal Gyrus	L	793	53.7	0.6	22.5	1.34	0.01	-3.18	56	-16	34
Middle Frontal Gyrus and Middle Orbital Gyrus	R	758	-26.8	-46.1	-6.0	1.30	0.02	-3.68	-38	-64	-12
Middle Occipital Gyrus	L	743	33.7	76.3	45.6	2.01	0.02	-4.11	6	76	52
Middle Frontal Gyrus	L	712	36.0	-44.9	-0.8	1.24	0.02	-2.70	40	-54	14
Cerebellum	L	549	3.3	40.0	-24.4	1.23	0.01	-2.62	0	44	-26

The model analyzed both conditions from the numerical Stroop task: Group + Exercise periods + Gender, and obtained the difference in activations between exercise periods in the Old and Young groups (ClusterSim corrected $p < 0.05$). The coordinate order is Right-Anterior-Inferior in the Montreal Neurological Institute (MNI) space. Volume = Size of each cluster volume; CM x = Center of mass (CM) for each cluster in the Right-Left direction; CM y = Center of mass for each cluster in the Anterior-Posterior direction; CM z = Center of mass for each cluster in the Inferior-Superior direction; Mean = Mean value for each volume cluster; SEM = Standard error of the mean for the volume cluster; Max Int = Maximum Intensity (peak) value for each volume cluster; Peak x = Peak value coordinate, R-L direction of each volume cluster; Peak y = Peak value coordinate, A-P direction of each volume cluster; Peak z = Peak value coordinate, I-S direction of each volume cluster.

Supplementary result 5. Regional values at different stages of exercise in the congruent condition.

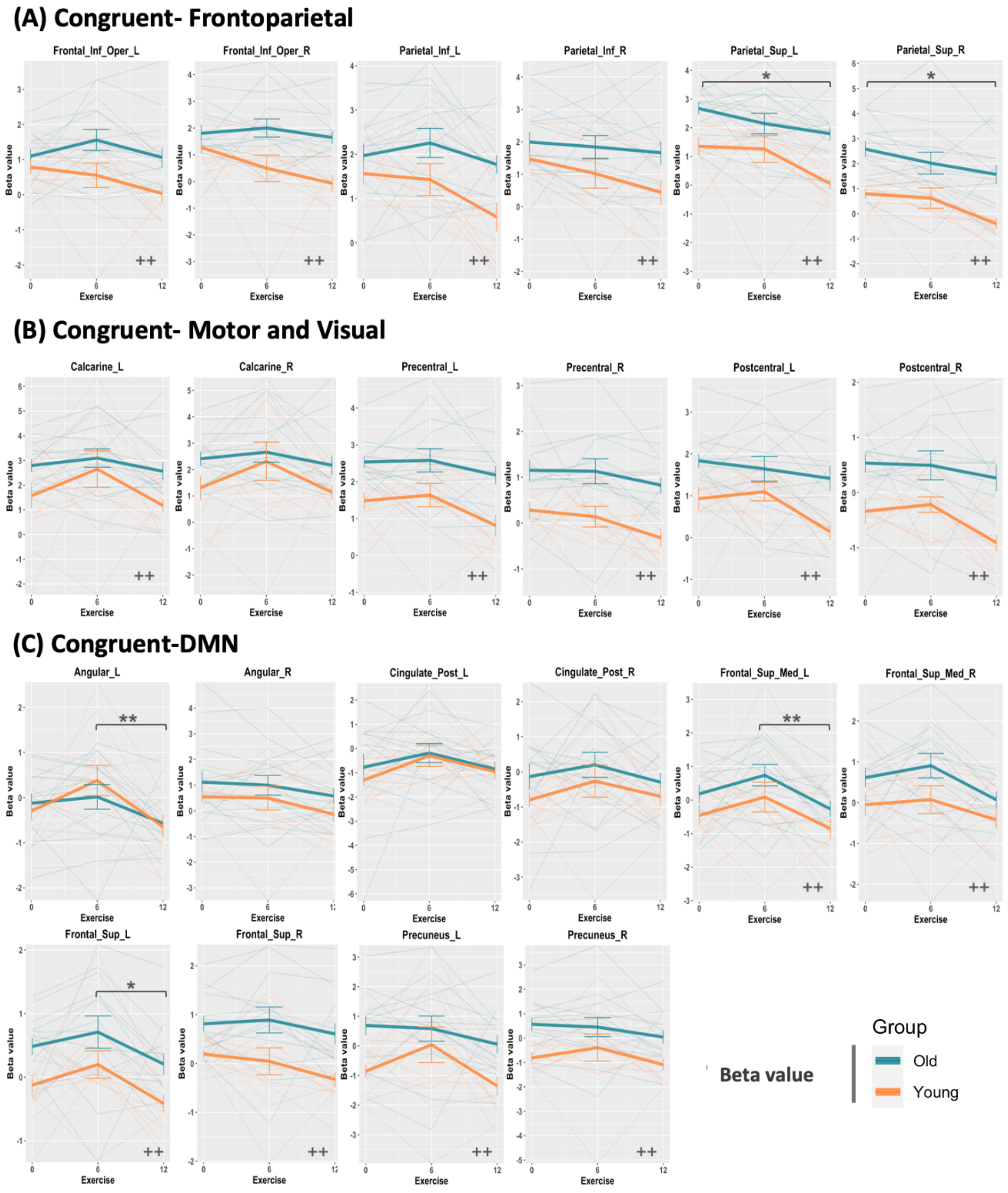

A-C are the fMRI signals of three times exercises in the congruent condition, which acquire regions of interest from automated anatomical labeling atlas 3 in frontoparietal, sensorimotor and visual, and default mode network (DMN). Green and orange lines are the change of Old and Young, respectively, and individual participant lines are presented in lighter colors. The statistical significance is calculated using the 2-way ANOVA, which factors are exercise periods and age group. The " * "means the p-value

is lower than 0.05 and higher than 0.01, and " ** " means the p-value is lower than 0.01 using the posthoc Bonferroni test in exercise periods. The " + "means the p-value is lower than 0.05 and higher than 0.01, and " ++ " means the p-value is lower than 0.01 using the posthoc Bonferroni test in the age group.

Supplementary result 6. Regional values at different stages of exercise in the incongruent condition.

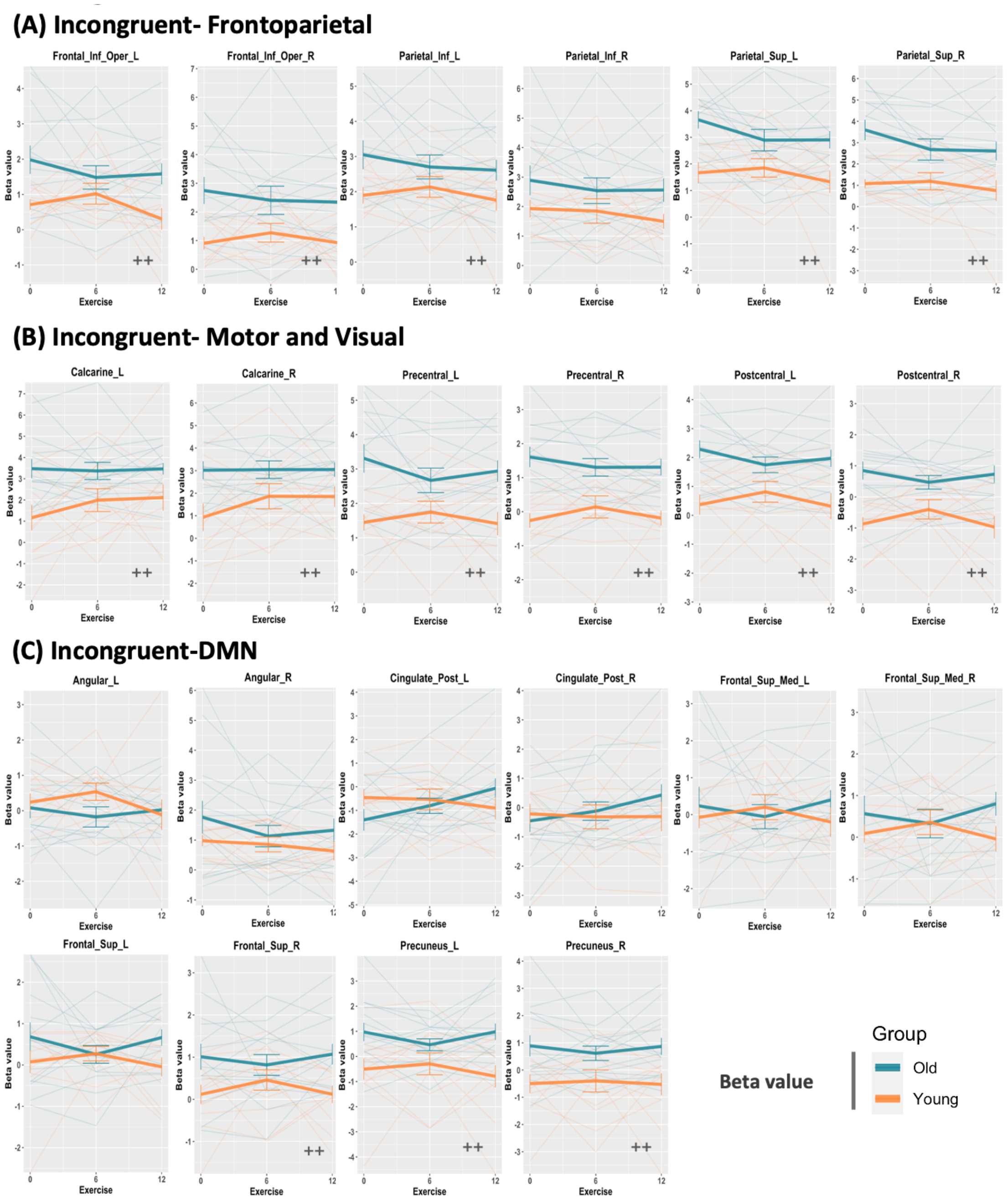

A-C are the fMRI signals of three times exercises in the incongruent condition, which acquire regions of interest from automated anatomical labeling atlas 3 in frontoparietal, sensorimotor and visual, and default mode network (DMN). Green and orange lines are the change of Old and Young, respectively, and individual participant lines are presented in lighter colors. The statistical significance is calculated using the 2-way ANOVA, which factors are exercise periods and age group. The " * "means the p-value is lower than 0.05 and higher than 0.01, and " ** " means the p-value is lower than 0.01 using the posthoc Bonferroni test in exercise periods. The " + "means the p-value is lower than 0.05 and higher than 0.01, and " ++ " means the p-value is lower than 0.01 using the posthoc Bonferroni test in the age group.

Supplementary result 7. The aging and exercise effects in the hippocampus.

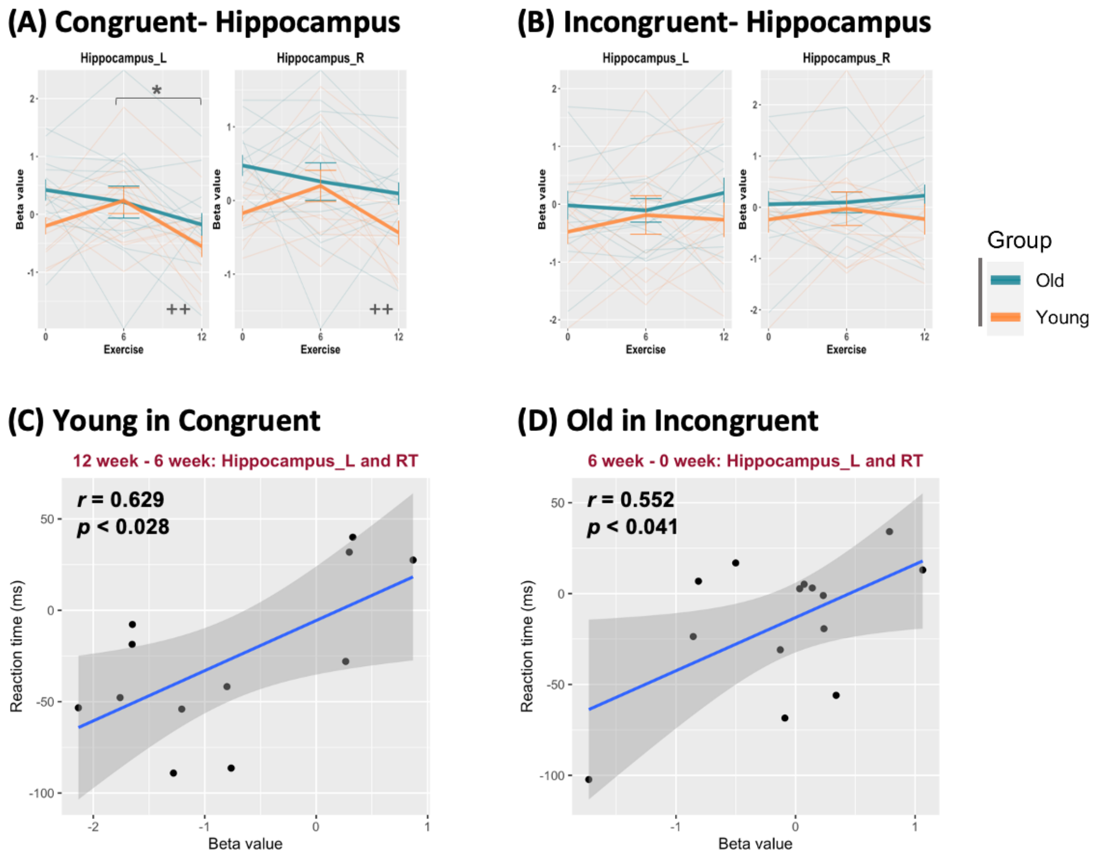

A and B are the fMRI signals of three times exercises in the congruent and incongruent condition, which acquire regions of interest from automated anatomical labeling atlas 3 in the hippocampus. Green and orange lines are the change of Old and Young, respectively. The statistical significance calculates using the 2-way ANOVA, which factors are exercise periods and age group. The " * "means the p-value is lower than 0.05 and higher than 0.01, and " ** " means the p-value is lower than 0.01 using the

posthoc Bonferroni test in exercise periods. The " + "means the p-value is lower than 0.05 and higher than 0.01, and " ++ " means the p-value is lower than 0.01 using the posthoc Bonferroni test in the age group. C and D are significant Pearson’s correlation results between changes in fMRI signals and reaction time for young and old adults.
